# Platelet-to-lymphocyte ratio is not a predictor of clinically significant prostate cancer at the prostate biopsy: A large cohort study

**DOI:** 10.1038/s41598-021-93637-3

**Published:** 2021-07-09

**Authors:** Jeong Woo Lee, Hyeon Jeong, Hwancheol Son, Min Chul Cho

**Affiliations:** 1 Department of Urology, Kyung Hee University Medical Center, Kyung Hee University College of Medicine, Seoul, Korea; 2 Department of Urology, Seoul Metropolitan Government-Seoul National University Boramae Medical Center, 20, Boramae-ro 5-Gil, Gongjak-gu, Seoul, 156-707 Korea

**Keywords:** Cancer, Urology

## Abstract

Previous studies have reported conflicting results on the predictive role of the platelet-to-lymphocyte ratio (PLR) in detecting clinically significant prostate cancer (CSPCa) at the time of prostate biopsies. We explored the predictive value of pre-biopsy PLRs for CSPCa using our large-cohort database. Consecutive men with serum prostate-specific antigen (PSA) levels of ≥ 3.0 ng/mL or abnormal digital rectal examination (DRE) findings and who underwent prostate biopsies were included in the study. There was no significant difference in the pre-biopsy PLR between men with benign disease, clinically insignificant prostate cancer (CISPCa), and CSPCa. Only the subset of CSPCa patients with serum PSA levels of < 10 ng/mL showed lower PLRs than those with benign disease or CISPCa. In the entire patient cohort, multivariate analyses revealed that older age, diabetes mellitus, DRE abnormalities, higher serum PSA levels, and smaller prostate volume were predictors of CSPCa. However, the pre-biopsy PLR was not a significant predictor of CSPCa at the prostate biopsy in the entire patient cohort or the subset of patients with serum PSA levels of < 10 ng/mL. In summary, the pre-biopsy PLR is not an independent predictor of CSPCa at the prostate biopsy, regardless of the serum PSA level.

## Introduction

Recently, prostate cancer (PCa) has been ranked as the second most frequent cancer and the fifth leading cause of cancer death in men^1^. For decades, the widespread use of prostate-specific antigen (PSA) screening has been associated with a significant decline in the incidence of advanced PCa and, thereby, a reduction in PCa-specific mortality^2^. However, there have been growing concerns about overdiagnosis and overtreatment because of the increased detection of clinically insignificant prostate cancer (CISPCa) in previously unscreened men^2,3^. Therefore, given that PSA is not cancer-specific and has a low positive predictive value of less than 25.0% for the detection of PCa^4^, there is a need for identifying useful markers as adjuncts to serum PSA levels for predicting clinically significant cancers, which thereby, can reduce the number of unnecessary prostate biopsies.

Under this background, magnetic resonance imaging (MRI), blood-based biomarkers (Prostate Health Index and the four-kallikrein score), urine-based biomarkers (PCA-3), positron emission tomography imaging using radiopharmaceuticals, and a few risk calculators (SelectMDx and the Stockholm-3 model) have been introduced to improve the accuracy of serum PSA levels in detecting clinically significant cancers before prostate biopsy^5,6^. However, their routine use before prostate biopsy cannot be generalized in real clinical practice due to limitations such as high cost, availability issues, and problems with insurance coverage. Thus, it is important to identify meaningful predictors of clinically significant cancers that are readily available in real clinical practice.

Recent evidence has suggested that inflammation is involved in tumorigenesis and the progression of PCa^7,8^. Also, a recent meta-analysis showed that systemic inflammatory markers such as the platelet-to-lymphocyte ratio (PLR), which could be easily calculated from the complete blood count (CBC), were associated with the prognosis of men with PCa^9^. In this context, a minority of studies of small or medium-sized cohorts have tested the predictive role of PLR in detecting clinically significant cancers at the time of prostate biopsies but reported conflicting results^10–14^. Therefore, we aimed to determine if the pre-biopsy PLR would predict clinically significant prostate cancers (CSPCa) at the time of the standard 12-core transrectal ultrasound-guided prostate biopsy (TRUS-Bx), currently the reference standard for the diagnosis of PCa, using our large cohort data.

## Materials and methods

### Subjects and research design

Between October 2010 and January 2020, a total of 1652 men who underwent standard 12-core TRUS-Bxs due to serum PSA levels of ≥ 3.0 ng/mL or abnormal DRE findings without the following exclusion criteria were included in the analysis of the present study. The exclusion criteria included retrieved biopsy cores of less than 12 and incomplete peri-biopsy data. The patients underwent pre-biopsy evaluations that included medical history, DRE, blood tests (CBC, blood urea nitrogen, creatinine, aspartate aminotransferase, alanine aminotransferase, prothrombin time, activated partial thromboplastin time, and PSA and testosterone levels), and urinalysis. The prospectively collected database was retrospectively analyzed in our study. The present study was approved by the SMG-SNU* Boramae Medical Center Institutional Review Board and followed the provisions of the Declaration of Helsinki (revised, Edinburgh 2000). This study is based only on retrospective analysis of clinical records of patients, and there is no minimum risk to patients during the study. Therefore, this study was exempted from obtaining informed consent after IRB review.

### Standard transrectal ultrasound-guided prostate biopsy and pathological data

The standard 12-core TRUS-Bx was performed in a routine manner, as described previously^15^. Briefly, after the patients laid on their side with their knees pulled up to their chest, they received local anesthesia using a periprostatic neurovascular bundle block. Biopsy tissue samples were collected using an automatic spring-propelled biopsy gun with an 18-G 22-mm core biopsy needle. A systematic 12-core biopsy scheme including the prostate apex and bilateral far lateral peripheral zones was used for the standard TRUS-Bxs. When some lesions on TRUS were suspicious of cancer, one or two biopsy samples were additionally collected.

The biopsy specimens were evaluated by uropathologists, and the Gleason grade groups or Gleason scores were determined according to the 2014 International Society of Urological Pathology classification (ISUP)^16^. The maximum percentage of cancer involvement and the number of biopsy cores positive for cancer were determined.

### Evaluation parameters and definitions

We defined clinically significant prostate cancer as Gleason grade groups of 2 or higher. The PLR was defined as the ratio of the platelet count to the absolute lymphocyte count, as follows: the platelet count/the absolute lymphocyte count. The prostate volumes on TRUS were measured tri-dimensionally in the following manner (the prolate ellipsoid formula): π/6 × transverse dimension (width) × anteroposterior dimension (length) × vertical dimension (height).

### Data analyses

Statistical comparisons between the continuous variables were performed using the independent t-test or the one-way analysis of variance (ANOVA) test for normally distributed data and the Mann–Whitney U test or the Kruskal–Wallis test for skewed data. For the categorical variables, statistical comparisons were performed using Fisher’s exact test or the χ^2^ test. To determine if PLR was a significant predictor of CSPCa, logistical regression analysis was performed with adjustments for explanatory variables such as age, body mass index (BMI), the presence or absence of diabetes mellitus (DM), the presence or absence of DRE abnormalities, serum PSA levels, serum testosterone levels, and prostate volume on TRUS. Statistical significance was defined as a p-value of < 0.05. The data analyses were conducted using SPSS Version 20.0 (SPSS Inc., Chicago, USA).

## Results

### Comparison of patient characteristics according to pre-biopsy serum PSA levels

Table 1 shows a comparison of the peri-biopsy data of the eligible patients according to serum PSA levels before the biopsy. The patients with higher serum PSA levels had older age, a higher rate of DRE abnormalities, a higher rate of prostate cancer or clinically significant cancer detection, a higher number of biopsy cores positive for cancer, and a greater maximum percentage of cancer infiltration in the biopsy cores that those with lower serum PSA levels. The patients with serum PSA levels of ≥ 20 ng/mL showed a higher prevalence of hypertension and lower serum testosterone levels compared to those with serum PSA levels of < 20 ng/mL and those with serum PSA levels of < 10 ng/mL, respectively. The patients with serum PSA levels of ≥ 10 ng/mL showed a higher pre-biopsy PLR and a larger prostate volume than those with serum PSA levels of < 10 ng/mL.

**Table 1:** Baseline characteristics of men who underwent standard 12-core TRUS-Bxs.

Variables	All patients (n = 1652)	PSA < 10 (n = 1072)	10 ≤ PSA < 20 (n = 344)	PSA ≥ 20 (n = 236)	p-value*
**Median (interquartile range) or number of patients (%)**					
Age, yr	68.0 (62.0–73.0)	66.0 (61.0–71.0)	69.0 (63.0–74.0)^†^	72.0 (66.3–76.0)^†‡^	< 0.001
BMI, kg/m^2^	24.04 (22.28–25.86)	24.16 (22.55–26.00)	23.86 (22.16–25.64)^†^	23.90 (21.45–25.77)	0.041
DM	282 (17.1%)	169 (15.8%)	64 (18.6%)	49 (17.1%)	0.126
HTN	711 (43.0%)	435 (40.6%)	150 (43.6%)	126 (53.4%)^††‡^	0.002
DRE abnormality	281 (17.0%)	123 (11.5%)	70 (20.3%)^†^	88 (37.3%)^†‡^	< 0.001
PSA, ng/mL	7.72 (5.07–13.39)	5.83 (4.26–7.52)	13.48 (11.34–16.24)^†^	43.70 (28.10–94.30)^†‡^	< 0.001
Serum testosterone, ng/mL	4.22 (3.36–5.56)	4.26 (3.41–5.66)	4.20 (3.49–5.54)	3.89 (2.94–5.56)^†^	0.058
Serum creatinine, mg/dL	0.92 (0.82–1.04)	0.92 (0.82–1.03)	0.94 (0.81–1.05)	0.92 (0.82–1.09)	0.479
Platelet-to-lymphocyte ratio	0.115 (0.093–0.145)	0.114 (0.093–0.141)	0.120 (0.092–0.153)^†^	0.118 (0.097–0.152)^†^	0.008
Total prostate volume, mL	38.0 (28.7–52.0)	37.0 (28.0–48.0)	42.0 (29.6–62.1)^†^	41.1 (30.7–57.9)^†^	< 0.001
Prostate cancer, GS grouping	540 (32.7%)	243 (22.7%)	125 (36.3%)^†^	172 (72.9%)^†‡^	< 0.001
GS 6 (3 + 3)	173 (10.5%)	123 (11.5%)	37 (10.8%)	13 (5.5%)	
GS 7 (3 + 4)	90 (5.4%)	40 (3.7%)	31 (9.0%)	19 (8.1%)	
GS 7 (4 + 3)	93 (5.6%)	41 (3.8%)	24 (7.0%)	28 (11.9%)	
GS 8	113 (6.8%)	29 (2.7%)	23 (6.7%)	61 (25.8%)	
GS 9 or 10	71 (4.3%)	10 (0.9%)	10 (2.9%)	51 (21.6%)	
Clinically significant prostate cancer	367 (22.2%)	120 (11.2%)	88 (25.6%)^†^	159 (67.4%)^†‡^	< 0.001
No. of positive cancer cores	4 (2–7)	3 (1–4)	4 (2–6)^†^	8 (6–11)^†‡^	< 0.001
Maximum percentage of cancer involvement in biopsy cores, %	43.4 (22.3–65.2)	44.8 (17.8–64.3)	37.5 (23.1–50.0)	70.6 (43.8–100.0)^†‡^	0.010

*BMI* body mass index, *DM* diabetes mellitus, *HTN* hypertension, *DRE* digital rectal examination, *PSA* prostate-specific antigen, *GS* Gleason score, *No.* number, *yr* years.

*p < 0.05: comparison of the variables between the groups using the Kruskal–Wallis test, the chi-squared test, or Fisher’s exact test, as indicated.

^†^p < 0.05: compared to the group with PSA < 10 using the Mann–Whitney U test, the chi-squared test, or Fisher’s exact test, as indicated.

^‡^p < 0.05: comparison between the groups with 10 ≤ PSA < 20 and PSA ≥ 20 using the Mann–Whitney U test, the chi-squared test, or Fisher’s exact test, as indicated.

### Only the subset of patients with serum PSA levels of < 10 ng/mL with clinically significant cancer had lower levels of PLR than those with clinically insignificant cancer or benign disease

A comparison of the explanatory variables between men with benign prostatic disease, clinically insignificant cancer, and clinically significant cancer is shown in Table 2. In the entire patient cohort, the patients with clinically significant cancer had older age, a higher rate of DRE abnormalities, higher serum PSA levels, and lower serum testosterone levels than those with clinically insignificant cancer or benign disease. However, there was no significant difference in the pre-biopsy PLR among the three groups. In a subset of patients with serum PSA levels of < 10 ng/mL, the patients with clinically significant cancer showed lower PLRs than those with clinically insignificant cancer or benign disease. In the subset of patients with serum PSA levels between 10 ng/mL and 20 ng/mL, the patients with clinically significant cancer showed lower serum testosterone levels and smaller prostatic volumes than those with clinically insignificant cancer or benign disease. However, there were no significant differences in pre-biopsy PLRs or serum PSA levels between the three groups. In the subset of patients with serum PSA levels of ≥ 20 ng/mL, the patients with clinically significant cancer showed a higher rate of DRE abnormalities and higher serum PSA levels than those with clinically insignificant cancer or benign disease. However, there was no difference in the pre-biopsy PLRs between the three groups.

**Table 2:** Comparison of explanatory variables between the patients with benign disease, clinically insignificant cancer, and clinically significant cancer.

All patients	Benign disease	Clinically insignificant cancer	Clinically significant cancer	p-value*
	(n = 1112)	(n = 173)	(n = 367)	
**Median (interquartile range) or number of patients (%)**				
Age, yr	66.0 (61.0–71.0)	69.0 (63.0–74.0)^†^	71.0 (67.0–76.0)^†‡^	0.001
Body mass index, kg/m^2^	24.0 (22.4–25.9)	24.3 (22.0–26.2)	23.9 (22.0–25.7)	0.243
Diabetes mellitus	171 (15.4%)	32 (18.5%)	79 (21.5%)^†^	0.022
Abnormal DRE finding	131 (11.8%)	25 (14.5%)	125 (34.1%)^†‡^	< 0.001
PSA, ng/mL	6.72 (4.54–10.07)	7.46 (5.61–10.77)^†^	16.9 (8.25–50.00)^†‡^	< 0.001
Testosterone, ng/mL	4.35 (3.50–5.68)	4.26 (3.39–5.54)	3.82 (2.90–5.24)^†‡^	0.027
Platelet-to-lymphocyte ratio	0.116 (0.094–0.144)	0.120 (0.096–0.147)	0.113 (0.091–0.147)	0.215
Total prostate volume, mL	41.0 (31.0–56.9)	32.0 (24.3–44.7)^†^	33.0 (25.0–43.0)^†^	0.928

Patients with PSAs < 10	Benign disease	Clinically insignificant cancer	Clinically significant cancer	p-value *
	(n = 829)	(n = 123)	(n = 120)	
**Median (interquartile range) or number of patients (%)**				
Age, yr	66.0 (61.0–71.0)	68.0 (62.0–73.0)^†^	69.0 (65.0–73.0)^†^	< 0.001
Body mass index, kg/m^2^	24.1 (22.5–25.9)	24.5 (22.7–26.5)	24.0 (22.6–25.8)	0.347
Diabetes mellitus	117 (14.1%)	24 (19.5%)	28 (23.3%)^†^	0.019
Abnormal DRE finding	81 (9.8%)	17 (13.8%)	25 (20.8%)^†^	0.002
PSA, ng/mL	5.53 (4.14–7.27)	6.44 (5.03–7.73)^†^	6.95 (5.42–8.17)^†^	< 0.001
Testosterone, ng/mL	4.31 (3.49–5.66)	4.16 (3.29–5.53)	4.07 (3.22–5.92)	0.478
Platelet-to-lymphocyte ratio	0.114 (0.093–0.141)	0.119 (0.094–0.144)	0.108 (0.086–0.127)^†‡^	0.046
Total prostate volume, mL	39.0 (30.1–51.2)	30.9 (23.0–39.9)^†^	30.2 (22.5–37.0)^†^	< 0.001

Patients with 10 ≤ PSA < 20	Benign disease	Clinically insignificant cancer	Clinically significant cancer	p-value *
	(n = 219)	(n = 37)	(n = 88)	
**Median (interquartile range) or number of patients (%)**				
Age, yr	67.0 (61.0–73.0)	72.0 (67.5–75.5)^†^	71.0 (67.0–76.0)^†^	< 0.001
Body mass index, kg/m^2^	23.8 (22.2–25.8)	23.5 (20.8–25.3)	23.9 (22.5–25.4)	0.572
Diabetes mellitus	43 (19.6%)	6 (16.2%)	15 (17.0%)	0.805
Abnormal DRE finding	38 (17.4%)	6 (16.2%)	26 (29.5%)^†^	0.045
PSA, ng/mL	13.56 (11.35–16.13)	13.38 (11.01–15.63)	13.39 (11.53–17.36)	0.441
Testosterone, ng/mL	4.50 (3.56–5.68)	4.44 (3.68–5.33)	3.79 (3.01–4.65)^†‡^	0.005
Platelet-to-lymphocyte ratio	0.119 (0.092–0.153)	0.120 (0.100–0.153)	0.121 (0.090–0.159)	0.806
Total prostate volume, mL	50.7 (33.0–73.0)	35.0 (29.8–57.3)^†^	30.1 (23.0–38.7)^†‡^	< 0.001

Patients with PSA ≥ 20	Benign disease	Clinically insignificant cancer	Clinically significant cancer	p-value *
	(n = 64)	(n = 13)	(n = 159)	
**Median (interquartile range) or number of patients (%)**				
Age, yr	69.5 (64.0–75.0)	72.0 (69.5–73.5)	73.0 (68.0–77.0)^†^	0.018
Body mass index, kg/m^2^	24.0 (21.5–26.0)	24.4 (21.6–24.9)	23.9 (21.4–25.8)	0.845
Diabetes mellitus	11 (17.2%)	2 (15.4%)	36 (22.6%)	0.695
Abnormal DRE finding	12 (18.8%)	2 (15.4%)	74 (46.5%)^†‡^	< 0.001
PSA, ng/mL	28.21 (22.47–37.23)	27.83 (22.36–39.28)	64.90 (37.20–149.00)^†‡^	< 0.001
Testosterone, ng/mL	4.69 (3.33–6.05)	4.45 (3.69–5.92)	3.80 (2.73–5.25)^†^	0.045
Platelet-to-lymphocyte ratio	0.124 (0.105–0.165)	0.116 (0.089–0.161)	0.115 (0.096–0.150)	0.329
Total prostate volume, mL	53.6 (37.0–82.3)	42.1 (24.7–58.2)^†^	38.9 (29.4–48.4)^†^	< 0.001

All variables were presented as median (interquartile range) or the number of patients (%).

*DRE* digital rectal examination, *PSA* prostate-specific antigen, *yr* years.

*p < 0.05: comparison of the variables between the groups using the Kruskal–Wallis test, the chi-squared test, or Fisher’s exact test, as indicated.

^†^p < 0.05: compared to the group with benign disease using the Mann–Whitney U test, the chi-squared test, or Fisher’s exact test, as indicated.

^‡^p < 0.05: comparison between the groups with clinically insignificant cancer and clinically significant cancers using the Mann–Whitney U test, the chi-squared test, or Fisher’s exact test, as indicated.

### Pre-biopsy PLR was not a predictor of clinically significant prostate cancer at the standard 12-core TRUS-Bx

Univariate and multivariate logistic regression analyses were performed to determine whether the pre-biopsy PLR predicted CSPCa at the standard 12-core TRUS-Bx. In the entire patient cohort, the logistic regression analysis showed that the pre-biopsy PLR was not a significant predictor of CSPCa at the prostate biopsy (Table 3). Instead, older age, the presence of DM, the presence of DRE abnormalities, higher serum PSA levels, and smaller prostate volume were predictors of clinically significant cancer. In the subset of patients with serum PSA levels of < 10 ng/mL, the multivariate regression model showed that older age, the presence of DM, higher serum PSA levels, and smaller prostate volumes were predictive factors of clinically significant cancer, whereas the pre-biopsy PLR was not. In the subset of patients with serum PSA levels between 10 ng/mL and 20 ng/mL, older age, lower serum testosterone levels, and smaller prostate volume were predictors of clinically significant cancers, whereas the PLR was not. In the subset of patients with serum PSA levels of ≥ 20 ng/mL, older age, the presence of DRE abnormalities, higher serum PSA levels, and smaller prostate volume were predictive factors of clinically significant cancers but the pre-biopsy PLR was not.

**Table 3:** Univariate and multivariate regression analyses to identify predictors of clinically significant prostate cancer (vs. benign disease + clinically insignificant prostate cancer) at the standard 12-core TRUS-Bx.

	Univariate	*p-value*	Multivariate	*p-value*
	OR (95% CI)		Adjusted OR (95% CI)	
**All patients**				
Age	1.100 (1.080**–**1.120)	< 0.001	1.102 (1.074**–**1.130)	< 0.001
BMI	0.960 (0.923**–**0.999)	0.046	1.023 (0.961**–**1.090)	0.469
Presence of DM	1.462 (1.093**–**1.955)	0.010	1.537 (1.013**–**2.331)	0.043
Abnormal DRE findings	3.738 (2.845**–**4.913)	< 0.001	1.891 (1.241**–**2.883)	0.003
Serum PSA	1.050 (1.041**–**1.060)	< 0.001	1.050 (1.038**–**1.062)	< 0.001
Serum testosterone	0.864 (0.797**–**0.938)	< 0.001	0.936 (0.848**–**1.033)	0.190
Total prostate volume	0.975 (0.968**–**0.982)	< 0.001	0.948 (0.937**–**0.960)	< 0.001
Platelet-to-lymphocyte ratio	0.600 (0.109**–**3.296)	0.557	0.094 (0.003–2.900)	0.176
**Patients with PSA < 10**				
Age	1.075 (1.044**–**1.107)	< 0.001	1.081 (1.042**–**1.122)	0.001
BMI	0.970 (0.909–1.036)	0.371	1.021 (0.929–1.123)	0.662
Presence of DM	1.751 (1.106–2.771)	0.017	1.972 (1.078–3.608)	0.027
Abnormal DRE findings	2.293 (1.408–3.734)	0.001	1.527 (0.762–3.060)	0.233
Serum PSA	1.238 (1.126**–**1.362)	< 0.001	1.313 (1.162**–**1.485)	< 0.001
Serum testosterone	1.003 (0.881–1.141)	0.967	1.054 (0.918–1.210)	0.454
Total prostate volume	0.945 (0.929–0.962)	< 0.001	0.934 (0.915–0.954)	< 0.001
Platelet-to-lymphocyte ratio	0.077 (0.001**–**7.058)	0.266	0.085 (0.000–22.774)	0.387
**Patients with 10 < PSA ≤ 20**				
Age	1.076 (1.039**–**1.116)	< 0.001	1.148 (1.08**–**1.214)	< 0.001
BMI	0.983 (0.901–1.072)	0.696	1.101 (0.965–1.256)	0.152
Presence of DM	0.868 (0.459–1.641)	0.663	0.716 (0.291–1.757)	0.465
Abnormal DRE findings	2.021 (1.153–3.542)	0.014	1.695 (0.737–3.900)	0.214
Serum PSA	1.055 (0.972**–**1.145)	0.197	1.112 (0.981**–**1.259)	0.096
Serum testosterone	0.724 (0.594–0.882)	0.001	0.761 (0.602–0.961)	0.022
Total prostate volume	0.945 (0.928–0.962)	< 0.001	0.929 (0.906–0.952)	< 0.001
Platelet-to-lymphocyte ratio	0.295 (0.006**–**13.600)	0.532	0.178 (0.000–112.526)	0.600
**Patients with PSA > 20**				
Age	1.054 (1.016–1.094)	0.005	1.081 (1.023–1.143)	0.006
BMI	0.985 (0.907–1.071)	0.727	1.016 (0.877–1.177)	0.830
Presence of DM	1.441 (0.714–2.909)	0.308	2.359 (0.741–7.512)	0.146
Abnormal DRE findings	3.918 (2.030–7.562)	< 0.001	3.325 (1.257–8.795)	0.015
Serum PSA	1.016 (1.008–1.025)	< 0.001	1.009 (1.002–1.016)	0.016
Serum testosterone	0.828 (0.694–0.988)	0.036	0.848 (0.676–1.065)	0.157
Total prostate volume	0.976 (0.965–0.988)	< 0.001	0.963 (0.945–0.982)	< 0.001
Platelet-to-lymphocyte ratio	0.036 (0.000–4.570)	0.178	0.004 (0.000–4.641)	0.123

*TRUS-Bx* transrectal ultrasound-guided prostate biopsy, *OR* odds ratio, *BMI* body mass index, *DM* diabetes mellitus, *PSA* prostate-specific antigen, *DRE* digital rectal examination.

## Discussion

The association between inflammation and cancer is still controversial. Most solid tumors have complex host-tumor relationships with inflammatory cells and mediators in the microenvironment^17^. In contrast, a review article on cancer immunology stated that inflammation and the immune system had inhibitory effects on carcinogenesis through tumor-related and tumor-specific antigen reactions^18^. A meta-analysis demonstrated that high PLRs, a marker of inflammation, led to the progression and poor prognosis of patients with various kinds of solid tumors, such as colorectal, hepatocellular, gastroesophageal, ovarian, and pancreatic cancer^19^. Platelets, perhaps through their ligands, interact with tumor cells or promote tumor cell adherence to the microvascular endothelium, allowing tumor cells to evade host immunosurveillance^20,21^. Lymphocytes regulate antitumor activity through host immune responses by inhibiting tumor proliferation and migration by secreting cytokines, such as TNF-α and INF-γ, and inducing cytotoxic apoptosis^22^. Based on these basic studies, it can be understood that a high PLR is associated with poor oncological outcomes in cancerous conditions. For PCa, a meta-analysis showed a significant correlation between high PLRs and poor overall survival in patients with localized PCa (HR = 1.72; 95% CI: 1.36–2.18, p < 0.001) but not in patients with metastatic castration-resistant PCa^9^. In a recent meta-analysis, patients with high pretreatment PLRs had higher cancer progression (HR = 1.62, 95% CI: 1.20–2.19, p = 0.002), overall mortality (HR = 1.70, 95% CI: 1.34–2.15, p < 0.001), and cancer-specific mortality (HR = 2.02, 95% CI: 1.24–3.29, p = 0.005) than those with low PLRs^23^.

Thus, useful markers as adjuncts to serum PSA levels are needed to avoid unnecessary biopsies or over-treatment while better predicting CSPCa prior to a TRUS-Bx, which currently is the standard modality for diagnosing PCa. Many studies have been conducted on the predictive role of inflammatory markers in the detection of CSPCa at the time of prostate biopsies, but conflicting results have been reported^10–14^. Our objective was to explore the role of pre-biopsy PLRs in predicting CSPCa at standard 12-core TRUS-Bxs in men suspected of PCa based on increased PSA levels or abnormal DRE findings using our large cohort dataset.

In a comparative study of Chinese populations^12^, the PLR was significantly higher in PCa patients than in normal men and patients with benign prostatic hyperplasia (BPH), and was an independent predictor of 3-year mortality in PCa patients. Similar results were reported in an Indonesian study, where significant differences were noted in the PLRs between PCa and BPH patients (p = 0.02)^11^. According to a study by Yuksel et al. in Turkish patients^14^, the PLRs were higher in PCa patients than in BPH patients (p = 0.018) but the PLR did not significantly differentiate patients with PCa from those with prostatitis. Murray et al.^13^ found that the pre-biopsy PLR was higher in PCa than in non-PCa patients (p = 0.048) but did not discriminate between CSPCa and CISPCa/benign disease at the initial biopsy in Chilean men with PSA levels of 4–10 ng/mL.

In contrast to these results, our comparative study using large-scale cohort data showed no significant difference in pre-biopsy PLRs among men with benign prostatic disease, CISPCa, and CSPCa at standard 12-core TRUS-Bxs in the entire patient cohort (p = 0.215). These results might be supported by a previous study analyzing the pathological results in men with PSAs between 4 and 10 ng/mL^10^, which found no significant difference in PLRs between men with benign lesions and those with PCa. There was a significant negative association between ISUP grades and PLRs (OR = -0.046; 95% CI: − 0.089 to − 0.003, p < 0.035)^10^. Our data showed that patients with CSPCa, defined as ISUP grade groups of ≥ 2, had significantly lower PLRs than those with benign disease or CISPCa in a subset of patients with serum PSA levels below 10 ng/mL (p = 0.046). A possible explanation for these results is that asymptomatic histological inflammation of the prostate is commonly found in men with BPH^24^. In this study, men with PCa had a significantly smaller prostate than those with benign disease. And it is thought that local inflammation might not be sufficient to alter the systemic markers of inflammation, such as the PLR.

From our results, older age, DM, DRE abnormalities, higher serum PSA levels, and smaller prostate volume were independent predictors of CSPCa at the standard 12-core TRUS-Bx in men with PSA levels of ≥ 3.0 ng/dL or abnormal DRE findings. In the subset of patients with serum PSA levels of < 10 ng/mL, older age, DM, higher serum PSA levels, and smaller prostate volume were predictive factors of CSPCa. In a study by Li et al.^10^, age and serum PSA levels were independent predictors and positively correlated with the pathological results in men with PSA levels between 4 and 10 ng/mL. According to these results, the pre-biopsy PLR was not a predictor of CSPCa at the standard 12-core TRUS-Bx in men with elevated PSA levels or abnormal DRE findings.

The present study had some limitations. The data were collected retrospectively with a non-randomized design. Second, the enrolled study cohort was a unique racial population. The incidence and prognosis of PCa in Koreans, which could be affected by genetics, environment, and lifestyle such as diet, are different from those of Western populations that may lead to mixed results.

## Conclusions

Based on the analyses of our large-scale cohort data, pre-biopsy PLRs did not differentiate men with benign prostatic disease from those with CISPCa or those with CSPCa at the standard 12-core TRUS-Bx with PSA levels of ≥ 3.0 ng/mL or abnormal DRE findings. Therefore, the pre-biopsy PLR, as an inflammatory marker, is not a significant predictor of CSPCa. Further large and prospective standardized trials are needed to determine the role of the PLR in predicting CSPCa at the prostate biopsy in men with clinically suspected PCa.
